# What Makes It Difficult to Start an Intimate Relationship: A Taxonomy of the Reasons

**DOI:** 10.5964/ejop.1852

**Published:** 2021-05-31

**Authors:** Menelaos Apostolou

**Affiliations:** a Department of Social Sciences, University of Nicosia, Nicosia, Cyprus; Department of Psychology, School of Social Sciences, Heriot Watt University, Edinburgh, United Kingdom

**Keywords:** intimate relationships, flirting, mismatch problem, mating, mate choice

## Abstract

Within the context of an evolutionary theoretical framework, the current research attempted to study the reasons that cause difficulties in starting an intimate relationship in the Greek cultural context. In particular, using qualitative research methods (interviews and open-ended questionnaires), Study 1 (N = 205) identified 58 reasons that make it difficult for people to start an intimate relationship. Using an online sample of 1,095 Greek-speaking participants (N = 1,095), Study 2 classified these reasons in 12 factors. More than 80% of the participants indicated that they faced above moderate or severe difficulties in at least one factor, while about 40% faced difficulties in three or more factors. Significant gender and age effects were found across the different factors. Using second order principal components analysis, the 12 factors were classified in three broader domains of difficulties in starting a relationship.

Many people are single, and the prevalence of singlehood in Western societies appears to be on the rise (Eurostat, 2015; Gallup, 2015; Jones, 2012). Recent research has indicated that one reason for this phenomenon is that people face difficulties in attracting a partner and initiating an intimate relationship (Apostolou, 2015; Apostolou, Shialos, Kyrou, Demetriou, & Papamichael, 2018). In the same vein, in the Greek-cultural context, it has been found that about one in two singles wanted to be in a relationship but they faced difficulties in doing so (Apostolou, Papadopoulou, & Georgiadou, 2018). The purpose of the current research is to identify the reasons that make it difficult for people to start a relationship. These reasons can be understood in an evolutionary theoretical framework that will be discussed first.

## The Evolutionary Roots of Poor Mating Performance

Humans are a sexually reproducing species, which means that, in order to reproduce, people need to gain access to the reproductive capacity of the opposite sex. In turn, this fact translates into strong evolutionary pressures being exercised on individuals to evolve mechanisms or adaptations that would enable them to gain such access (Buss, 2017). To put it differently, those who are not able to gain such access do not have any offspring and thus, their genetic material is less likely to be represented in future generations. Accordingly, we have inherited from our ancestors mechanisms that serve this purpose. For instance, emotions, such as romantic love and sexual desire, are adaptations that motivate people to divert their energy in obtaining mates (Apostolou, 2016b). Similarly, mate preferences are adaptations that enable individuals to divert their attention to prospective mates with good reproductive potential (Buss, 1989; Iwasa & Pomiankowski, 1994).

Selection forces adjust the frequency of the genes in the gene pool, so that individuals that compose a given population are endowed with mechanisms that make them well-adapted to the environment they occupy. When a certain aspect of the environment changes, selection forces operate on the genes that code for traits interacting with this aspect of the environment, so as to adjust their frequencies to the demands of the new environment (Irons, 1998). This process takes time, and usually, several generations are required before the population adapts to these new conditions (Irons, 1998; Weiner, 1995). During this time, several individuals will have mechanisms (e.g., adaptations involved in flirting) that are not well adapted to the prevailing conditions, and as a consequence, they will suffer fitness penalties. This is known as the mismatch problem: Mechanisms do not work well, not because they are broken but because they have evolved to function in an environment which is different from the environment they currently have to function in (Crawford, 1998; Li, van Vugt, & Colarelli, 2017; Maner & Kenrick, 2010). The number of individuals that would be affected by the mismatch problem and the severity of the fitness penalties they would suffer depend on the recency and the extent of the environmental change.

In particular, the more distant in the past the change has been, the more time selection forces would have had to adjust the frequencies of the alleles in the gene pool to these conditions, and the less likely it would be for contemporary people to carry alleles that are not optimal for these conditions. Moreover, if the environmental change has not been extensive, the mechanisms people are endowed with may still be able to function reasonably well. However, if the change has been considerable, these mechanisms may not be able to function at all. Therefore, the bigger the change, the more fitness penalties individuals who carry non-optimal alleles would suffer. Overall, a considerable and recent change in a specific aspect of the environment is likely to have a substantial negative impact on many individuals. We will argue below that such a change has occurred in the domain of mating (Apostolou, 2019; Crawford, 1998; Li et al., 2017).

## Mismatch in the Mating Domain

Men form male coalitions in order to fight other men and monopolize their resources, including women, by force (Ghiglieri, 1999; Tooby & Cosmides, 1988; Trivers, 1985). These conflicts take the form of small-scale raids, but they can also escalate to full scale wars. Anthropological, historical and archeological evidence indicates that such conflicts were frequent in ancestral societies (Bowles, 2009; Keegan, 2004; Keeley, 1996). Furthermore, anthropological evidence from pre-industrial societies, along with evidence from historical records, indicates that in ancestral human societies mating was regulated (Apostolou, 2007, 2010, 2012; Broude & Green, 1983; Coontz, 2006). In particularly, young individuals were controlled by their parents, especially their fathers, who would choose spouses for them (Apostolou, 2007, 2010). Anthropological and historical evidence indicates further that individuals had also a space to exercise mate choice, even where arranged marriage was prevalent (Apostolou, 2017a; Coontz, 2006).

On the basis of the available evidence, we can argue that in ancestral human societies, and during most of the human evolutionary time, mating took place in a context where marriages were arranged, fights with reproductive purposes were common, and individuals had a limited but significant space to attract mates on their own. To put it differently, ancestral individuals could get mates from their parents or through force or through being able to persuade opposite-sex individuals to mate with them. Following the industrial revolution in the 18th century, and the subsequent transition to post-industrialism in the 20th century, this aspect of the environment changed drastically; fights and wars became much less frequent, while people were constrained by law and other social institutions from forcing sex from desirable others (Pinker, 2011). Parents in Western societies lost most of their power to control mate choice directly, and they could only do so indirectly through manipulation (Apostolou & Papageorgi, 2014). As a consequence, individuals became free to choose their own mates. But, although more freedom is desirable, many people were confronted with the mismatch problem: They had to find mates on their own, and to do so they had to rely on mechanisms which have not been calibrated by selection forces for accomplishing this task (Apostolou, 2015).

In particular, our ancestors would predominantly secure mates from their parents or through force; thus, their adaptations have been optimized for this purpose and not for attracting mates on their own. People living in post-industrial societies have inherited these mechanisms, but these mechanisms cannot help them, as mating in the contemporary environment is not regulated by parents and it is not forced by means of male coalitions. Actually, some of these mechanisms that would result in high reproductive success in the ancestral context, may have the opposite effect in the contemporary one. For instance, high aggression in an ancestral environment may have turned a man to be a successful warrior who managed to monopolize access to many women, but in the modern environment such high aggression would most likely turn a man to an undesirable mate (Apostolou, 2016a).

Overall, the drastic change in the environment would lead to many individuals having mechanisms which are inadequate for attracting mates in the contemporary context. The number of these individuals is expected to be high, because this change occurred very recently in evolutionary terms. For example, many Western societies completed the transition to post-industrialism only a few generations ago (Simms, 2014).

## Difficulties in Starting an Intimate Relationship

The theoretical framework developed above leads to the prediction that a considerable proportion of individuals living in post-industrial societies would face difficulties in intimate relationships. Some older studies found that people remain single for different reasons, including personal deficits and self-blame (Austrom & Hanel, 1985). Frazier Arikian, Benson, Losoff, and Maurer (1996) asked 251 men and women in the USA why they were not married. They found that one of the most common reasons was “difficulty in establishing a relationship,” although they did not investigate what caused this difficulty. Furthermore, in a study performed using a sample of 160 women in India, Prabhakar (2011) found that one reason for being single was poor health or disability.

More recently, one study based on a sample of 1,894 participants (1,114 women, 780 men) found that, about one in two individuals experienced difficulties in either starting or keeping a relationship (Apostolou, Shialos et al., 2018). It was also found that poor performance in mating was predicted by sexual functioning, self-esteem, self-perceived mate value, choosiness, personality, attention to looks, and mating effort, which were attributed to the mismatch problem. However, the predictors of mating performance were chosen on theoretical grounds, rather than on what people have indicated to prevent them from doing well in mating. A different study employed qualitative research methods in order to identify the reasons that drive individuals to be single, and subsequently, it employed quantitative research methods in order to classify these reasons to broader categories (Apostolou, 2017b). Nevertheless, this research focused on singlehood in general rather than on the reasons that make it difficult for an individual to start a relationship. Thus, although some of the reasons for the latter are included in the former, this study did not provide a comprehensive account of these reasons. Finally, a recent qualitative study analyzed 13,429 responses from a Reddit thread asking the question why men were single (Apostolou, 2019). The responses were classified in 43 broader categories, with the most frequent ones being poor flirting skills, low self-confidence, poor looks, shyness, low effort, and bad experience from previous relationships. This research was limited however to men, while it did not examine specifically the factors that prevented people from initiating an intimate relationship.

In sum, to our knowledge, there is no study that has attempted to examine the reasons that cause difficulties in initiating an intimate relationship. The current study attempts to fill in this gap in the literature by identifying the difficulties that make it hard for individuals to attract mates. Please note that the current study employed a mixed research design used in previous studies (e.g., Apostolou, 2017b).

## Study 1

The purpose of this study was to identify the difficulties that people face in attracting mates. We employed a combination of qualitative research methods, namely, in-depth interviews and open-ended questionnaires. More specifically, although the in-depth interview method can provide rich information about the reasons which cause difficulties in starting a relationship, participants may not be willing to entrust the interviewer with all reasons, especially if these are of sensitive nature, such as sexual problems (Liamputtong, 2009). Therefore, we have also employed the open-ended questionnaire method, where participants could respond to questions while maintaining their anonymity.

### Method

#### Participants

Participation was on a voluntary basis, and no compensation was given. The main requirement for taking part was to be an adult (18 years old or older). Participants were recruited through an advertisement that was placed on social media and through word of mouth. There were two groups of participants, a group responding to interviews and a group responding to open-ended questionnaires. Starting with the in-depth interviews, 61 Greeks and Greek-Cypriots took part (32 women, 29 men). The mean age of women was 25.2 (*SD* = 6.9), and the mean age of men was 26.6 (*SD* = 7.4). Moreover, 40.5% of the participants were single, 25.4% were in a relationship, 25.1% were married and 9% were divorced. With respect to the open-ended questionnaires, 144 Greeks and Greek-Cypriots took part (71 women, 73 men). The mean age of women was 23.7 (*SD* = 8.3), and the mean age of men was 25.5 (*SD* = 8.1). Furthermore, 42.0% of the participants were single, 32.6% were in a relationship, 20.0% were married and 5.4% were divorced.

#### Materials

##### In-Depth Interviews

The research took place in a medium-sized private university in the Republic of Cyprus. In order to identify the different reasons that caused difficulties to individuals in starting a relationship, a series of semi-structured interviews were conducted. All interviews took place in a psychology lab located at the university premises. The interviews were in Greek and each interview lasted about 35 minutes. The interviews were conducted by one of the authors and one independent graduate student. At the beginning of the interview, participants were asked to sign a consent form, and subsequently, they were subsequently asked to fill in their demographic details (i.e., gender, age, marital status).

Participants were asked to discuss the different reasons that led them to face currently or in the past difficulties in starting a relationship. Participants were also asked to discuss difficulties that their friends and relatives were having in starting a relationship. Follow-up and probing questions were used in order to get more detailed information on specific difficulties. The participants’ answers were recorded on paper. At the end of the interview, participants were debriefed and thanked.

##### Open-Ended Questionnaires

The survey had two parts. In the first part, participants were asked to indicate different factors that caused them difficulties in starting an intimate relationship now or in the past. In the second part, demographic information was collected (i.e., gender, age, marital status).

### Results

Two independent graduate students, a man and a woman, processed participants’ answers for the in-depth interviews and the open-ended questionnaires. Each assessed the answers independently, and they generated a list of the reasons reported by the participants. For this purpose, redundant reasons with identical or very similar wording were eliminated. We followed this procedure in order to produce an instrument of a reasonable length which would not include redundancies. Answers that contained multiple reasons were eliminated, as they were difficult to interpret. In addition, difficulties with unclear or vague wording were also eliminated. If there was disagreement about retaining a given item, this was resolved by consulting one of the authors. In total, 58 reasons that caused difficulties in starting an intimate relationship were identified and are presented in Table 1.

**Table 1:** The Extracted Factors and the Respective Factor Loadings for Study 2

Factor	Factor loading
Poor courtship skills	
Flirting causes me stress	.798
I do not know what to say when I want to approach someone	.778
I find it difficult to approach someone	.773
I am not good at flirting	.769
I do not take the initiative to approach someone I like	.754
I find it difficult to put myself in the process of flirting	.739
I do not know how to show to someone that I am interested	.738
I am shy	.668
I find it difficult to figure out if someone is interested in me	.536
The fear of rejection	.395
I do not know how to start a relationship	.350
I am socially awkward	.280
I am introverted	.278
Fear of commitment	
Fear of commitment	.840
I do not like commitment	.815
I fear that I will lose my freedom	.801
I am not sure whether I want to be in a relationship	.735
I do not feel ready to be in a relationship	.692
I do not want trouble	.541
The effort and the time it takes for a relationship discourages me	.474
Fear of getting hurt	
I fear that I will get hurt	.810
I fear that I will be disappointed	.768
The fear of breaking up	.703
I have bad experiences from previous relationships	.579
Health/handicap issues	
I have a health problem	.825
I have a handicap	.808
I have a sexual issue	.368
I have a psychological issue	.312
Difficult to meet available mates	
It is not easy for me to meet people who are available as mates	.586
My way turns away potential mates	.580
I am clingy	.511
I do not have a social circle with potential mates	.468
People do not show an interest in me	.398
Lack of time	
Lack of time	−.804
I do not have any free time to look for a relationship	−.775
Too conservative	
My religious beliefs	.644
My family is restricting me to make a relationship	.634
I am very conservative	.612
I fear that I will be socially stigmatized	.539
Lack of money	
I do not have enough money to support a relationship	−.903
I am in a poor financial condition	−.899
Poor looks and low self-confidence	
My appearance	−.765
My body weight	−.726
I do not consider myself desirable	−.556
I feel insecure about whether others will like me	−.448
I am insecure	−.426
I have low self-confidence	−.423
I have not achieved much in my life and I think I am not a desirable mate	−.350
My character	−.325
Sexual orientation	
My sexual orientation (I am homosexual)	−.640
Too picky	
I am not easily interested in someone	.764
I am too picky	.747
It is hard to find mates that make a good match with me	.619
I always find faults in potential partners	.505
I am not easily giving opportunities to people who approach me	.444
I do not trust easily	.381
I am easily disappointed	.372
Not get over previous relationship	
I have not got over my previous partner	−.674

## Study 2

The purpose of this study was to classify the difficulties identified in Study 1 to broader difficulties in starting an intimate relationship.

### Method

#### Participants

The research was designed and ran in a private university in the Republic of Cyprus. The study was performed online. We chose this method because of the sensitive nature of the research, since we could get more honest answers this way (Kreuter, Presser, & Tourangeau, 2008). The link of the study was forwarded as a Facebook add. The target group was set to men and women who were 18 years old or older.

In this study, 1,095 Greek-speaking individuals took part (590 women, 505 men). The mean age of women was 26.68 (*SD* = 9.07) and the mean age of men was 29.73 (*SD* = 10.01). Moreover, 67.4% of the participants were single, 19.9% were in a relationship, 9.0% were divorced, and 3.7% were married. Please note that the demographic information of the current study has been also used by Apostolou et al. (2018), in estimating the occurrence of singlehood in the Greek cultural context.

#### Materials

The survey was constructed using Google forms, and consisted of two parts. In the first part, participants were given the 58 reasons identified in Study 1, and were asked to rate how each one is currently causing or has caused them in the past difficulties in starting an intimate relationship, using a five-points Likert scale (1- strongly disagree, 5- strongly agree). The order of presentation of the difficulties was randomized across participants. Finally, in the second part demographic information was collected (gender, age, marital status).

### Results

In order to classify the different reasons that caused difficulties in starting a relationship into broader categories, principal components analysis was performed, with direct oblimin as a rotation method. The results suggested a 12 factor solution (Eigenvalue > 1). The Kaiser-Meyer-Olkin (KMO) statistic was .92, indicating a very good sample adequacy. The scales produced were checked by means of reliability analysis (Cronbach’s α). Internal consistency (α) ranged from .63 to .88 with a mean of .76. The factor structure is presented in Table 1.

The first factor that emerged was the “Poor courtship skills,” which related to the difficulties that individuals had in approaching a partner and initiating courtship. Participants indicated that they were not good at flirting, that they were shy and that they experienced fear of rejection. Participants indicated also that they would not take the initiative approaching prospective mates even if they liked them. Another facet of this factor was that participants indicated a difficulty in understanding whether someone was interested in them. We can also see that being socially awkward and introverted loaded to this factor albeit weakly.

The next factor was the “Fear of commitment,” where participants indicated that having a fear of or that not liking commitment, made it difficult for them to start a relationship. Here, participants indicated that they feared that they would lose their freedom, and that the time and effort that a relationship involved discouraged them from being in one. In the “Fear of getting hurt,” participants indicated fear of getting hurt and of being disappointed as reasons that kept them back from starting a relationship. Having bad experiences from previous relationships loaded also in this factor, suggesting that participants had been hurt in the past, and feared that it can happen again in the future. In the “Health/handicap issues,” participants indicated that a health problem or a handicap prevented them from being in a relationship. Having a sexual or a psychological problem loaded also in this factor, albeit more weakly.

In the “Difficult to meet available mates,” participants indicated that they faced difficulties in meeting available mates. One facet of this factor was that participants’ manner turned away potential mates. In the “Lack of time,” people indicated that they did not have sufficient time to allocate in finding mates and in keeping a relationship. In the “Too conservative,” participants indicated that their conservatism, their religious beliefs and their family prevented them from starting a relationship. Moreover, in the “Lack of money,” people indicated that they did not have enough money to support a relationship. In the “Poor looks and low self-confidence,” participants indicated that their looks and body weight prevented them from being in a relationship. Self-confidence and being insecure, loaded also high in this factor, suggesting that poor looks had a negative effect on self-confidence which in turn, affected people’s capacity to initiate an intimate relationship.

In the “Sexual orientation,” participants indicated that being homosexual prevented them from being in a relationship. Furthermore, in the “Too picky,” reasons such as being very choosy, not being easily interested in a mate, not giving opportunities to prospective mates, finding faults and not trusting others, constrained people from starting a relationship. Finally, in the “Not get over previous relationship,” participants indicated that not getting over a past relationship constrained them from starting a new one.

In order to obtain an estimate of importance in causing difficulties to individuals, we calculated means and standard deviations for each factor. The results are presented in Table 2, where we can see that “The fear of getting hurt,” “Too picky,” “Difficult to meet available mates” and “Poor courtship skills” topped the hierarchy. In the middle of the hierarchy, were factors such as “Poor looks and low self-confidence” and “Lack of time,” while at the bottom of the hierarchy were the “Health/handicap issues” and the “Sexual orientation.”

**Table 2:** Significant Gender and Age Effects for the 12 Extracted Factors in Study 2

Factor	Rank	Overall		Women		Men		*p*	$\eta_{p}^{2}$	Age^a^	
		*M*	*SD*	*M*	*SD*	*M*	*SD*			*p*	$\eta_{p}^{2}$
Fear of getting hurt	1	3.16	1.08	3.36	1.03	2.93	1.10	< .001	.033	(−) .001	.019
Too picky	2	3.15	0.81	3.27	0.78	3.01	0.82	< .001	.060	n.s.	n.s.
Difficult to meet available mates	3	2.96	0.86	2.87	0.88	3.06	0.84	< .001	.046	(+) < .001	.043
Poor courtship skills	4	2.78	0.94	2.65	0.91	2.93	0.95	< .001	.102	(−) .003	.032
Poor looks and low self-confidence	5	2.52	0.88	2.48	0.88	2.57	0.87	< .001	.066	(−) < .001	.050
Lack of time	6	2.13	1.18	2.28	1.22	2.28	1.15	n.s.	n.s.	n.s.	n.s.
Lack of money	7	2.30	1.19	2.12	1.13	2.51	1.23	< .001	.022	(+) < .001	.023
Fear of commitment	8	2.24	0.94	2.29	0.96	2.18	0.92	.001	.024	(−) < .001	.037
Not get over previous relationship	9	2.17	1.41	2.19	1.41	2.18	1.40	n.s.	n.s.	n.s.	n.s.
Too conservative	10	1.70	0.72	1.70	0.73	1.70	0.71	< .001	.026	(+) < .001	.038
Health/handicap issues	11	1.39	0.55	1.35	0.51	1.43	0.61	n.s.	n.s.	(+) < .001	.021
Sexual orientation	12	1.22	0.78	1.20	0.70	1.29	1.16	n.s.	n.s.	n.s.	n.s.

^a^The sign of the coefficient of age was placed in parenthesis.

We would also like to know how many participants faced above average or severe difficulties in each domain. For this purpose we calculated the percentages of individuals who had a mean score of 3.5 or more (as the middle of the scale was the “3,” it is reasonable to assume that scores above this number would indicate above average agreement that this is a problem they face difficulty) in each factor. The results are presented in Table 3, where we have also estimated the percentages of men and women separately. We can see, for instance, that about one in three men and one in five women experienced the difficulty of poor courtship skills. We estimated further that 17.2% did not face any above average or severe difficulty, which means that 82.8% faced at least one. More specifically, 23.4% faced one, 20.2% two, 15.9% three, 9.4% four, 7.5% five and 3.8% six, while the remaining 2.6% faced seven or more difficulties. Finally, we estimated that those who were single faced on average 2.48 (*SD* = 1.95) difficulties, those in a relationship 1.97 (*SD* = 1.76), those married 1.88 (*SD* = 1.45) and those divorced 1.82 (*SD* = 1.48).

**Table 3:** The Percentage of Participants Facing Above Average or Severe Difficulties in Each Factor

Factor	%			*p*	OR^ab^
	Total	Women	Men		
Fear of getting hurt	45.9	54.1	36.3	< .001	0.51 (1.96)
Too picky	33.7	39.9	26.6	< .001	0.51 (1.96)
Difficult to meet available mates	27.4	23.9	31.5	n.s.	n.s.
Poor courtship skills	25.3	19.8	31.8	< .001	2.08 (0.48)
Poor looks and low self-confidence	17.2	15.8	18.9	n.s.	n.s.
Lack of time	22.0	21.4	22.7	n.s.	n.s.
Lack of money	20.9	15.8	26.9	< .001	1.91 (0.52)
Fear of commitment	10.95	12.1	9.6	n.s.	n.s.
Not get over previous relationship	20.0	20.2	19.6	n.s.	n.s.
Too conservative	3.1	3.2	2.8	n.s.	n.s.
Health/handicap issues	1.5	1.2	1.8	n.s.	n.s.
Sexual orientation	3.9	2.7	5.2	.004	2.48 (0.40)

*Note*. OR = odds ratio.

^a^Men in relation to women, the reference category is the “below 3.5 mean score.” We have estimated in parenthesis the odds ratio for the reference category being “3.5 mean score or more.”

^b^The reference category is the “I am between relationships—I prefer it this way.”

#### Contingencies

In order to estimate significant effects, we ran a series of Multivariate Analysis of Covariance (MANCOVA) tests, where the reasons composing each factor were entered as the dependent variables and the participants’ gender and age were entered as the independent variables. For the “Sexual orientation” and the “Not get over previous relationships,” which involved only one reason, we ran Analysis of Covariance (ANCOVA) tests instead. Overall, we ran 12 tests, one for each factor. To avoid the problem of alpha inflation arising from multiple comparisons, we employed Bonferroni correction, and we set the alpha to .004 (.05/12).

The results are presented in Table 2, where we can see that for most of the factors significant gender and age effects emerged. As indicated by the effect sizes, the largest gender difference was found for the “Poor courtship skills,” where men gave higher scores than women, followed by the “Poor looks and low self-confidence,” where men also gave higher scores than women. In the latter factor, for the “my body weight” reason, women gave higher score than men but the difference did not pass the significance level. Third in the hierarchy was the “Too picky,” where women gave higher scores than men. Moreover, for the too conservative, the means overlapped, but the result came significant predominantly due to the significant difference for the “My family is restricting me to make a relationship,” where women gave significantly higher scores (*M* = 1.69, *SD* = 1.16) than men (*M* = 1.46, *SD* = 0.97). With respect to age, the strongest effect was on the “Poor looks and low self-confidence,” where younger participants gave higher scores than older ones. It was followed by the “The difficult to meet available mates” and the “Too conservative,” where older participants gave higher scores than younger ones.

Furthermore, we attempted to estimate significant differences in the frequencies of above average difficulties calculated above. For this purpose, we ran a series of binomial logistic regressions, where participants’ classification (3.5 mean score or more, below 3.5 mean score) was entered as the dependent variable, and participants’ gender and age were entered as the independent variables. This analysis was performed for each factor. Bonferroni correction for alpha inflation was applied as above. The results are presented in Table 3, where we can see that men were more likely to classify as “3.5 mean score or more” for “The poor courtship skills,” “Lack of money” and “Sexual orientation” than women. On the other hand, women were more likely to classify as “3.5 mean score or more” for the “Fear of getting hurt” and the “Too picky.”

#### Second Order Factor Structure

We would like to investigate whether the 12 factors identified above could be classified in broader domains. For this purpose, we applied principal components analysis, with direct oblimin as a rotation method, on the 12 factors. The results suggested a three factor solution (Eigenvalue > 1). The KMO statistic was .79, indicating a good sample adequacy. The scales produced were checked by means of reliability analysis (Cronbach’s α). Internal consistency (α) ranged from .61 to .75 with a mean of .67. The factor structure is presented in Table 4.

**Table 4:** The Domains Derived From Applying Second Order Principal Components Analysis in Study 2

Domain	Factor loading
Low capacity for courtship	
Poor courtship skills	.872
Poor looks and low self-confidence	.853
Difficult to meet available mates	.747
Too conservative	.378
Fear of getting hurt	.337
Unwillingness to compromise	
Too picky	.776
Fear of commitment	.726
Lack of time	.702
Lack of money	.232
Constraints	
Sexual orientation	.805
Health/handicap issues	.624
Not get over previous relationship	.381

The first domain that emerged was named “Capacity for courtship,” since the factors that loaded to it were associated with courtship including flirting skills, capacity to approach a partner and being desirable to prospective partners. We interpreted the second domain to reflect unwillingness to make compromises in order to be in a relationship. From the factors that loaded in this domain, we can see for instance that, participants were very picky and unwilling to lower their standards in order to get a mate. Also, the lack of money and time could be interpreted to indicate that participants were unwilling to commit these resources in the mating effort. For example, individuals would allocate their limited time in endeavors, such as working, and would be unwilling to compromise their career in order to make time for a relationship. Similarly, in the “Fear of commitment,” participants may not want to compromise their freedom for the shake of a relationship. Finally, we interpreted the third domain to reflect “Constraints,” as participants having health problems or handicaps, being homosexual and not having got over a past relationship, constrained them from starting a relationship.

In order to assess importance, we estimated the means and the standard deviations for each domain, and we placed them in a hierarchical order. As we can see from Table 5, at the top of the hierarchy was the “Capacity for courtship,” followed by the “Unwillingness to make compromises” and the “Constraints.”

**Table 5:** Significant Gender and Age Effects for the Three Extracted Domains

Domain	Rank	Overall		Women		Men		*p*	$\eta_{p}^{2}$	Age	
		*M*	*SD*	*M*	*SD*	*M*	*SD*			*p*	$\eta_{p}^{2}$
Low capacity for courtship	1	2.62	0.63	2.61	0.62	2.63	0.65	< .001	.089	< .001	.079
Unwillingness to compromise	2	2.50	0.74	2.49	0.70	2.52	0.70	< .001	.068	< .001	.028
Constraints	3	1.60	0.64	1.58	0.61	1.62	0.67	n.s.	n.s.	n.s.	n.s.

#### Gender and Age Effects

In order to investigate gender and age effects, we ran a series of MANCOVAs, where the factors composing each domain were entered as the dependent variables and the gender and the age were entered as the independent variables. The analysis was performed separately for each domain, and the results are presented in Table 5. We can see that significant gender and age effects were found for the “Capacity for courtship” and for the “Unwillingness to make compromises.”

## Discussion

In the current research, we identified 58 reasons, which clustered in 12 factors that caused difficulties to individuals in starting an intimate relationship. More than 80% of participants indicated that they faced above moderate or severe difficulties in at least one factor, while about 40% faced difficulties in three or more factors. Significant gender and age effects were also found across the different factors. Using second order principal components analysis, the 12 factors were classified in three broader domains of difficulties in starting a relationship. The low capacity for flirting was the domain in which participants exhibited the most difficulties.

Starting from the latter finding, it appears that the primary difficulty that prevented people in our sample to initiate a relationship, was a limited capacity to approach prospective mates and initiate flirting. This capacity was compromised by poor flirting skills, shyness, low confidence coming from worries about looks, not meeting available mates, but also by being too conservative and worrying that they would get hurt. We argue that the high prevalence of this difficulty is predominantly due to the mismatch problem: Until very recently, in ancestral pre-industrial societies, individuals would get access to mates through force or through arranged marriage, and they would not have to gain such access through initiating flirting. As a consequence, many people did not inherit from their ancestors a good flirting capacity, which is required for getting access to mates in a contemporary context, where mating is not regulated or forced.

With respect to poor flirting skills, a gender difference was found, which was the largest one across the 12 factors, with men indicating more severe difficulty than women. One possible reason is that due to the asymmetry in parental investment, it is usually men who take the initiative to approach women (Buss, 1989, 2017; Trivers, 1972). Thus, flirting skills are more important to men, and so lacking such skills may cause them more severe difficulties. Another reason can be that, in ancestral societies men gained access to women through force (Ghiglieri, 1999; Tooby & Cosmides, 1988), and they have evolved to be aggressive and rough, which would facilitate them to do so. Yet, such traits are impairing when a man tries to initiate flirting.

Moving on, finding an intimate partner involves adjusting mating standards to realistic levels and use resources such as time and effort effectively. In the ancestral pre-industrial context, a great part of the choosing was made by parents, so mechanisms involved in mating effort and selection of mates may not have been properly adjusted to work well in a context where individuals have to find mates on their own. As a consequence, several people today may have unreasonably high standards, which drive them toward mates they can never get, rejecting mates that they could get. In addition, they may devote their energy in endeavors, such as making money, leaving little time for seeking mates.

This domain does not reflect only the mismatch problem, but also the optimal functioning of mechanisms that have evolved to facilitate mate choice. To begin with, being choosy may lead to people rejecting several mates before settling with one, having to stay single for some time. Nevertheless, because settling with the first mate that comes in someone’s way, is unlikely to be optimal—such a mate may lack desirable traits—being choosy constitutes an effective mechanism that can lead to better and more long-lasting intimate relationships. Thus, many people may tend to interpret being picky as a constraint from starting a relationship, which actually constrains them from starting a relationship with no prospects, giving them time to look for one which has prospects.

Similarly, because people are choosy, it would pay for mate-seekers to develop the qualities which are considered desirable before entering in the mating market. For instance, being educated, having a good job, and being financially independent, are highly desirable in the mating market (Buss, 2017; Buss et al., 1990), but require considerable resources, such as money and time, in order to be developed. Accordingly, one possible beneficial strategy would be to allocate these resources in developing these qualities instead of seeking mates, and to enter in the mating market at a later time, having better chances of success. In the meantime, people may not have sufficient time, money or willingness to start a relationship, which albeit constraining in starting a relationship now, may be enabling in starting one at a future time.

This argument can be seen also in the significant gender difference in the “Too picky” factor which loads in this domain. Women gave significantly higher scores than men, suggesting that pickiness made it more difficult for them to start a relationship. Yet, due to the risk of pregnancy, a sexual encounter can commit a woman’s parental investment to a man who is unwilling to invest to her and to her children, a risk that men do not face. Women have evolved to be choosier than men as a way to protect them from such a risk (Buss, 2017). Consequently, women are likely to reject more potential mates than men before settling with one, resulting in more time being single, and so to be more likely to report pickiness as a reason that keeps them back from starting a relationship.

The “Constraints” domain appeared to be the least important one, preventing individuals from starting a relationship. This is expected, as severe health problems and handicaps are rare. In addition, health problems in particular arise usually at an older age, where individuals are not active mate-seekers. Moreover, being homosexual was another constraining factor. In Study 1, individuals indicated that being homosexual prevented them from starting a relationship because they were in the closet, or had difficulties in meeting other homosexual individuals. Yet, the prevalence of homosexuality is the population is about 5% (LeVay, 2010), which can explain why this was not a frequent reason in our sample.

The current research is not without limitations. To begin with, our sample is not representative of the population; for instance, single individuals are overrepresented. One possible reason is that those who were single would be more interested in participating in our study. In addition, our research was based on self-report data, and participants may not have had an adequate understanding of the reasons that caused them difficulties in starting a relationship. Furthermore, to our knowledge, this is the first study that has attempted to study the reasons that cause difficulties in starting a relationship. From the results of a single study, we cannot be certain neither that the factor structure we have derived here is the true one nor that we have identified all the reasons that cause difficulties in starting a relationship. Considerable replication and extension of the current work is required, in order to understand the reasons which prevent people from staring a relationship.

Replication in different cultural contexts is also necessary, because our findings are based on the Greek culture, and may not readily apply to other cultures. In particular, the factor structure and the importance assigned to each factor may differ across cultural settings. For instance, in cultural settings where marriages are arranged, the flirting capacity is unlikely to be the most important factor preventing people from starting an intimate relationship. On the other hand, in cultural settings where people find their own partners, the flirting capacity would an important factor preventing people from staring an intimate relationship.

In sum, the current study identified several reasons which constrained people from starting an intimate relationship. These reasons were classified in broader factors and domains, and sex and age effects were also found. Considerable more work is necessary however, in order to fully understand this complex phenomenon.
